# Using machine learning to investigate self-medication purchasing in England via high street retailer loyalty card data

**DOI:** 10.1371/journal.pone.0207523

**Published:** 2018-11-19

**Authors:** Alec Davies, Mark A. Green, Alex D. Singleton

**Affiliations:** Geographic Data Science Lab, Department of Geography & Planning, University of Liverpool, Liverpool, United Kingdom; University of Missouri Kansas City, UNITED STATES

## Abstract

The availability alongside growing awareness of medicine has led to increased self-treatment of minor ailments. Self-medication is where one ‘self’ diagnoses and prescribes over the counter medicines for treatment. The self-care movement has important policy implications, perceived to relieve the National Health Service (NHS) burden, increasing patient subsistence and freeing resources for more serious ailments. However, there has been little research exploring how self-medication behaviours vary between population groups due to a lack of available data. The aim of our study is to evaluate how high street retailer loyalty card data can help inform our understanding of how individuals self-medicate in England. Transaction level loyalty card data was acquired from a national high street retailer for England for 2012–2014. We calculated the proportion of loyalty card customers (n ~ 10 million) within Lower Super Output Areas who purchased the following medicines: ‘coughs and colds’, ‘Hayfever’, ‘pain relief’ and ‘sun preps’. Machine learning was used to explore how 50 sociodemographic and health accessibility features were associated towards explaining purchasing of each product group. Random Forests are used as a baseline and Gradient Boosting as our final model. Our results showed that pain relief was the most common medicine purchased. There was little difference in purchasing behaviours by sex other than for sun preps. The gradient boosting models demonstrated that socioeconomic status of areas, as well as air pollution, were important predictors of each medicine. Our study adds to the self-medication literature through demonstrating the usefulness of loyalty card records for producing insights about how self-medication varies at the national level. Big data offer novel insights that add to and address issues that traditional studies are unable to consider. New forms of data through data linkage may offer opportunities to improve current public health decision making surrounding at risk population groups within self-medication behaviours.

## Introduction

The economic health-care burden of minor ailments (e.g. coughs and colds, sunburn) on the National Health Service (NHS) is extensive [1]. Self-care, a globally adopted movement, empowers patients to take control of their healthcare [2,3]. Self-medication occurs via over-the-counter (OTC) medicines used to treat minor ailments. Patients assume a greater health management responsibility as they diagnose and select suitable medical treatment, which can reduce the burden on health care providers. This process is typically hybridised with advice from health care professionals or online services such as WebMD [4].

Traditionally OTC products are weaker than medicines available through prescription, although increasingly medication is becoming available at pharmacies via deregulation [4–6]. OTC and Prescription (Rx) can be identical medication, however, the main differences are cost to patient and pack size [7,8]. OTC pharmaceuticals have purchase quantity restrictions and therefore are typically used for short term treatment [4,9]. Cost is influential for medication route as some population groups in England are Rx fee exempt (e.g. elderly, pregnant). Cheap or weak medication prescription costs have witnessed scrutiny with paracetamol highlighted as a high cost to the NHS [10]. It is possible that social factors such as poverty or income could influence the likelihood of self-medication.

Despite the benefits to the health-care industry, self-care may result in mistreatment of medications which could have severe consequences and increased burden [4]. Consultation of ailments between patients and clinicians may be lacking within self-care dependence [2,4,11]. Delay of treatment or misdiagnosis, concurrent medication and unrelated medical conditions cause increased risk during self-medication [9]. Side effects due to additional health complications (and other behaviours such as alcohol consumption) can be serious particularly if products are not correctly labelled or if patients are not medication literate [11–13]. Accidental and purposeful poisoning creates a considerable issue to the NHS with paracetamol related poisonings accounting for 15% of total poisonings [11]. Pain killers are most liable for abuse from OTC drugs [14]. Developing effective population surveillance systems to identify potential harms represents an important yet difficult venture.

The self-care movement is somewhat fuelled from smart devices and fitness tracking [15]. Health records are increasingly digitised [16], and smart cities increasingly commonplace [17]. Data linkage across health care would allow practitioners greater awareness of patient medication to reduce the risk of side effects [18,19]. People are greater informed via WebMD, longer clinic hours and video appointments, meaning diagnosis is the most accessible it has ever been. New forms of data and applied methods to deal with these data mean more can be known, showing an importance and relevance of applied big data research.

New forms of (big) data are non-traditional data sources collected for purposes other than research (e.g. loyalty card records, social media profiles, smart sensors) and are increasingly available to health researchers [20]. One of these new forms of data, loyalty card records, offers interest to researchers and policy makers. Traditional research that has explored how self-medication behaviours vary throughout the population have only utilised self-reported data from health surveys [21]. Self-reported data has been shown elsewhere to be affected by bias [21] and objective purchasing behaviours may offer one solution for minimising such bias. Such data are often ‘big’ and cover national scales, compared to smaller health surveys that are often localised to smaller regions and therefore have less relevance to the national scale where public health policy decision making is often made. They also offer a less intrusive form of data collection since data are collected routinely by organisations. Real time purchase information for minor ailment medicines may be useful for improving surveillance systems (particularly through data linkage).

The aim of this study is to investigate how high street retailer data can help to inform our understanding of how individuals self-medicate in England.

## Materials and methods

### Data sources

The outcome data explored in this study is transaction records linked to customer loyalty cards provided from a national high street retailer. The primary use of loyalty cards is to increase customer knowledge and thus strengthen retailer loyalty [22]. When a customer purchases a product and provides a loyalty card their transaction is logged against their account in return for incentives and promotions. When customers register for a loyalty card, they are asked to provide additional details including age, gender and address.

Data were provided as individual transactions for ~ 300 categories of products. Upon accessing the data, this was cleaned from 15 million to 10 million customers by age, gender and postcode. The cleaning process was required to account for unrealistic ages or missing data. All non-England postcodes were removed due to differences in how prescribed medicines are funded between countries of the UK.

Transactions were aggregated by customer and product group to determine whether a customer purchased a product within the two-year period, April 2012 to 2014. The product groups were *coughs and colds* (e.g. cough suppressants, throat lozenges), *Hayfever* (e.g. antihistamines), *pain relief* (e.g. paracetamol, ibuprofen) and *sun preps* (e.g. sun lotions). These categories were the lowest level and most detailed aggregation available. This allowed for a comparison between OTC medicines whilst maintaining as much detail as possible. Higher aggregations were provided in a hierarchy but using these would mean a loss of self-medication context (e.g. sun preps would be grouped as ‘toiletries’). This information was aggregated for Local Authority District (LAD) and Lower Super Output Area (LSOA) using National Statistics Postcode Lookup [23], and converted to the proportion of total customers per geography. LAD level (n = 326) was used as this is the lowest level allowed to publish data spatially by the data provider; LSOA (n = 32844) was used in our analytical models to provide more detailed spatial resolution of our sociodemographic predictors.

We selected a diverse range of sociodemographic explanatory variables to explore how they related to self-medication patterns. These were selected based on previous research that has demonstrated that multiple aspects of an individual’s social circumstances are associated with their likelihood of consuming self-medicines [12,21]. The objective was to utilise many sociodemographic variables as no single variable can best measure any social issue, as well as leveraging the machine learning approach that can handle a large number of features. Explanatory (variables) included Output Area Classification (OAC) [24], Rural Urban Classification (RUC) [25], Index of Multiple Deprivation (IMD) [26] and Index of Access to Healthy Assets and Hazards (AHAH) [27]. OAC groups were used to measure population characteristics and were aggregated to LSOA level using proportions of each group. IMD score was used to account for deprivation. AHAH was included as it comprises a range of measures of health-related environmental features such as air quality and accessibility to health care [27]. All the variables can be seen in the S1 Table.

### Statistical analysis

Exploratory analysis was performed to understand national level patterns using the LAD level aggregated data. We calculated the overall distribution of purchasing each product stratified by gender to examine which medicines were most common. We then mapped overall purchasing patterns to explore how behaviours varied geographically.

A machine learning approach was applied to explore important sociodemographic characteristics of purchasing patterns for self-medication products. The rational was the effectiveness of these statistical methods in capturing data complexity via scalable learning systems for the utilisation of large data [28]. Non-parametric modelling allows analysis of large numbers of observations and measures that require better predictive models in feasible time frames [6]. Scaling up models such as general linear models is possible, but typically falls short in predictive power. Machine learning, in particular tree based models, fit a richer class of functions enabling the exploitation of data and are widely applied and highly effective particularly in ensemble methods [28]. Various feature types as well as large feature and sample sizes can be utilised as each feature is treated separately.

Two regression tree ensemble methods were applied. The first, Random Forests, is a tree ensemble method that fits a piecewise constant surface over the domain by recursive partitioning, in a greedy fashion–constantly improving [6]. Variables are selected automatically from a subset which adds the ‘randomness’. Random Forests are accurate out of the bag requiring little hyperparameter tuning. The method was selected as the baseline for model performance. The randomness prevents model overfitting, and the method is robust to noise as it selects strong complex learners with low bias [29].

Boosting and in particular Extreme Gradient Boosting (XGBoost) was the second tree ensemble method selected. Boosting combines weak classifiers to produce an ensemble classifier with superior generalised misclassification of error [29]. Boosting resamples training points giving more weight to misclassified points boosting for performance in problematic areas of feature space. This is repeated to produce a stream of classifiers combined through voting to produce the overall classifier [6]. Hyperparameter tuning is fundamental to boosting can significantly change the model, however this brings greater computational complexity to produce better performance [6]. Hyperparameter tuning is strict towards overfitting. The key difference is Random Forests is focused on reducing bias, whereas XGBoost reduces variance to build a model. XGBoost is used as the method we are most interested in due to the increased performance that comes from hyperparameter tuning, whilst the parallel application allows greater computational complexity in shorter time frames.

The four self-medication product groups were used: *coughs and colds*, *Hayfever*, *pain relief* and *sun preps*. Random Forests and XGBoost models for each product class were created. Data for each product contained n = 32844 records. These data were split into 70% training datasets (n = 22993). The remaining 30% (n = 9851) was used as holdout datasets (unseen test datasets) to assess model performance. The unit of analysis are LSOAs (n = 32844).

Random Forests have few hyperparameters to tune, hence the reputation for being a very accurate out of the bag learning method. The column subsample (number of features) for each tree was 1/3, and the number of trees (rounds) was constrained to 500 as there with little gain of extending above this. The model was utilised as ‘out of the bag’ with default settings.

Contrastingly as hyperparameter tuning is very important for XGBoost, hyperparameters were found using an aggressive grid search to find the best combination within the range provided. The grid search included 10-fold cross-validation allowing for optimal hyperparameters to be found for each model (shown Table 1). Random Forests computation time was greater than XGBoost; XGBoost uses shallower tree depth and a parallel computing implementation. Model performance is analysed using performance metrics of R2 and RMSE. Feature importance ranking is used to compare feature selection across model types, and partial dependency plots are used to explore the relationship between the most important features and the outcome variables of proportional product purchase. Despite machine learning algorithms witnessing performance increase, context is often lost. Partial dependency plots are similar in function to coefficients in OLS regression, allowing for context to be retained [30]. Partial dependency plots hold all variables constant within the model except the specified variable which is varied across its range. The allows interpretation of how the target variable changes as the specified variable changes, capturing correlations.

**Table 1:** Comparison of machine learning model performance.

	Coughs and colds		Hayfever		Pain relief		Sun preps	
	Random Forests	XGBoost	Random Forests	XGBoost	Random Forests	XGBoost	Random Forest	XGBoost
Training sample size	70%	70%	70%	70%	70%	70%	70%	70%
*Hyper-parameters*								
Learning Rate		0.01		0.01		0.01		0.01
Gamma		0		0		0		0
Minimum child weight		1		1		1		1
Column subsample	.33	.7	.33	.7	.33	.7	.33	.7
Row subsample		.8		.8		.8		.8
Maximum depth		6		6		6		6
Rounds	500	5000	500	5000	500	5000	500	5000
*Performance*								
R2	.5030	.5010	.5881	.5993	.6010	.6063	.6148	.6379
RMSE	.0492	.0493	.0391	.0388	.0427	.0423	.0475	.0460
Run Time (minutes)	10	2	10	2	10	2	10	2

Learning rate = step size shrinkage used to make model conservative; Gamma = minimum loss reduction to make further partition; Minimum child weight = minimum instance weight needed in a child; Maximum depth = maximum depth of a tree (number of splits) [28]; RMSE = Root Mean Squared Error

Random Forests were created in the randomForest R package [31], gradient boosting in the XGBoost R package [32] and the data splits, hyper parameter search and model evaluation was performed using the caret R package [33]. The ‘pdp’ r package [30] was used to explore the marginal effect of the top 5 ranked features.

## Results

### Overall purchasing behaviours

**Fig 1:**
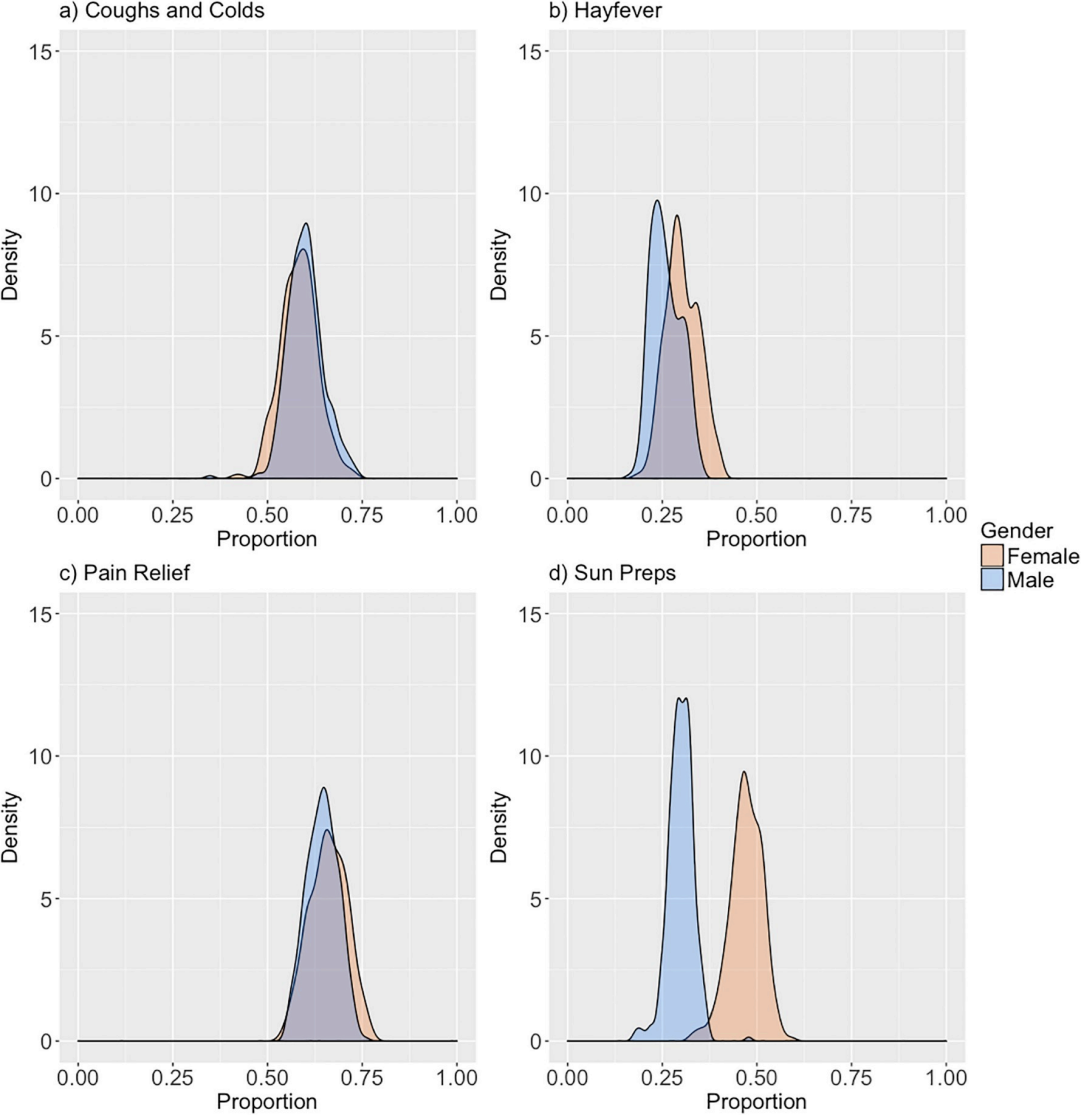
Proportion per local authority level of self-medication products by gender.

Fig 1 shows each of the product group proportion distributions by gender. Pain relief is shown to have the highest proportion of purchasing (median of 65.94%), whereas Hayfever the lowest (median of 29.41%). One explanation for why Hayfever has lower purchasing than the other products is that the associated condition does not affect the whole population. Pain Relief and coughs and colds (median 65.84% and 58.56%) both have high purchasing proportion due their relative high accessibility in England, in part related to how common they are as ailments [34,35]. Each of the products have similar distributions for both males and females, suggesting there isn’t gender sensitivity within loyalty card customers for these product groups. Sun preps purchasing is the only product with a significant difference in the distribution, with proportions almost double for females (median 29.98 male, 47.01% female).

**Fig 2:**
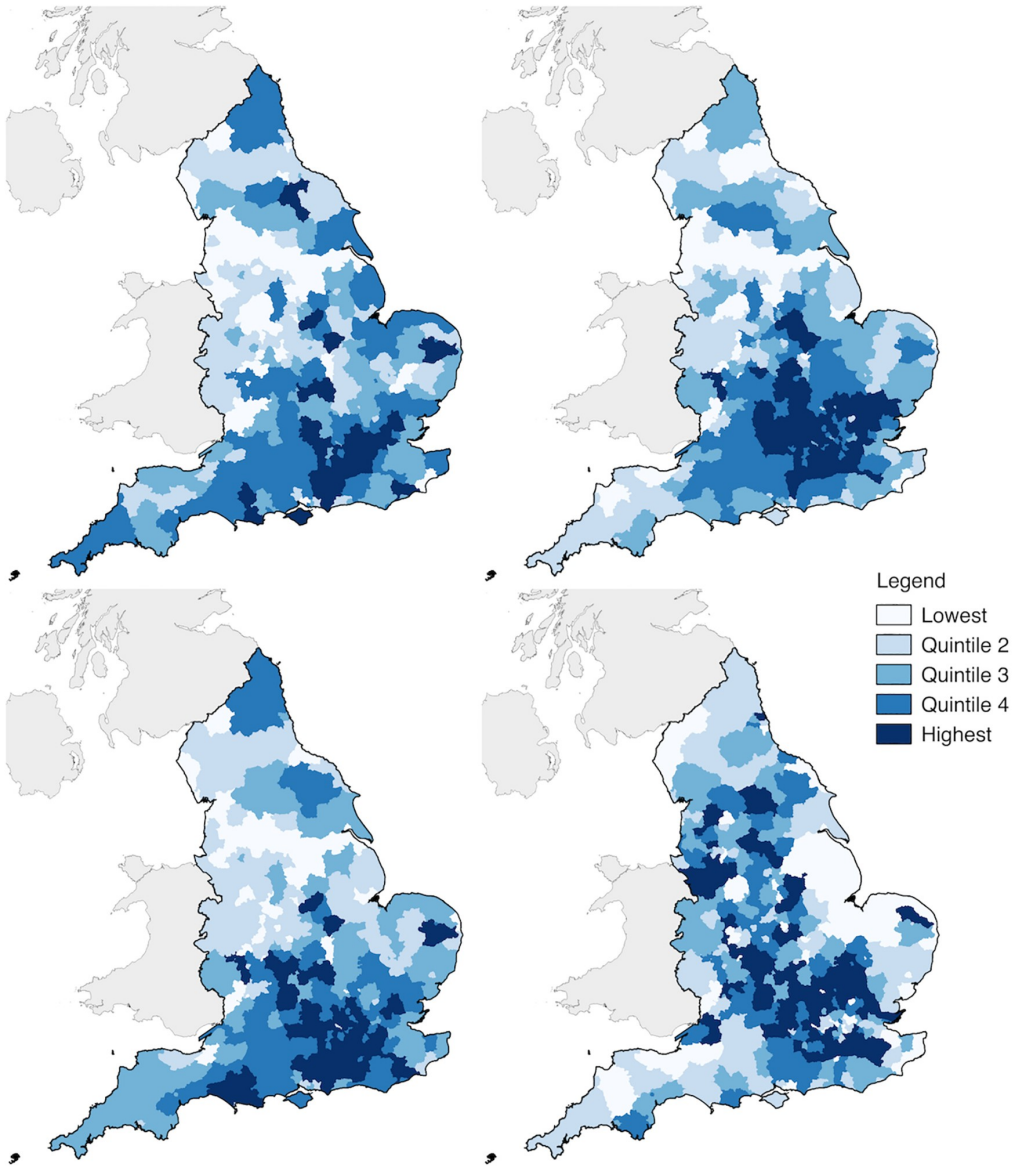
Proportion per local authority level of self-medication products. (Top left *coughs and colds*, top right *Hayfever*, bottom left *pain relief*, bottom right *sun preps*).

Fig 2 plots the geographic variation in purchasing of each product by quintiles at Local Authority District (LAD) level. A consistent spatial pattern of higher purchasing in London and the South-East region is observed for each product bar sun preps. For coughs and colds, Hayfever and pain relief there are distinct North-South differences with the North-West regions exhibiting lower purchasing. Sun preps exhibit a differing spatial pattern from the other medicines, with urban and central areas displaying higher proportion of sales compared to costal and rural areas (e.g. East Anglia and the South-East).

### Explaining sociodemographic correlates of purchasing behaviours

Table 1 shows the performance metrics of Root Mean Squared Error (RMSE) and *R*^2^ for each of the models. XGBoost performs better for both metrics except for coughs and colds where the performance is marginally worse (.002 worse for *R*^2^, .0001 for RMSE). Sun preps has the best predictive performance, however this product group exhibits the greatest variance between performance metrics with Random Forests performing .0231 worse with for *R*^2^. Despite the poorest performance being for coughs and colds at .5010, there is good predictive performance across all our models. The difference in predictive ability shows that of the variables included the variance is explained for some products more than others. Further variables may be included if the goal was solely predictive performance.

**Fig 3:**
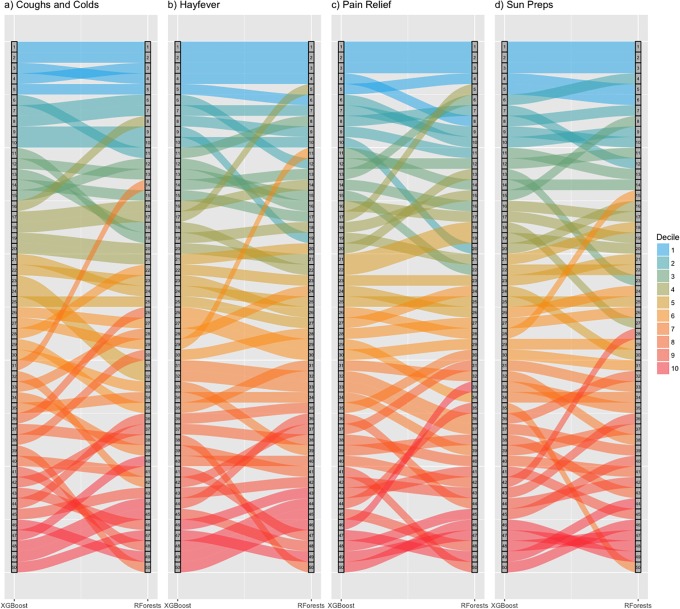
Rank comparison of feature importance. (Note: ‘Decile’ refers to the decile of ranks from XGBoost).

The models purpose is to investigate which sociodemographic factors are important for predicting purchasing patterns. We focus on the top 5 most important features from each model as these have the highest influence on overall model performance, with the remaining variables having less impact. The top 5 variables account for as much as 50% of loss reduction in the models. To visualise feature importance, we use Alluvial plots (an extension of Sankey diagrams) to show how ranks vary between models for each medicine. Fig 3 shows the ranks coloured by decile. The highest feature importance is stable for each medicine, showing similar features are consistently important for both methods. There is greater variability seen further down the variable rankings where variables have smaller effects.

**Fig 4:**
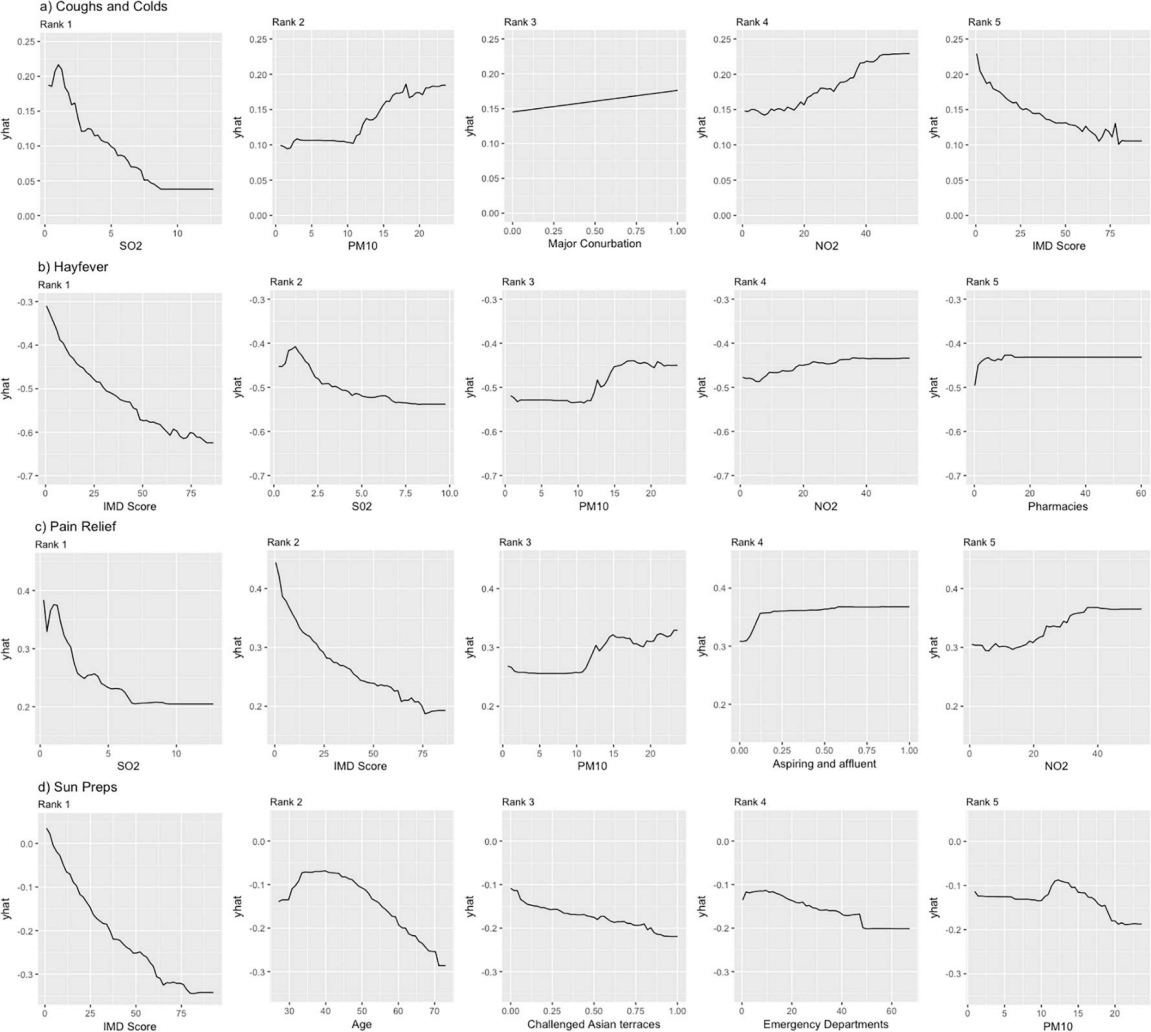
Partial dependency plots. (Note: top 5 products from each XGBoost model).

Fig 4 presents the partial dependence plots for each model of the top 5 most important variables. A yhat value of 0 (y axis) represents the average proportion of customers. Positive values are interpreted as an increase, negative a decrease from the average value.

There are three broad patterns observed. Socioeconomic features were stable and commonly high ranking in each model, particularly the IMD score (9 of 20 occurrences). Areas that had higher IMD scores were negatively associated with purchasing patterns. Air quality variables were also common (10 out of 20). Particulate matter (PM10) and nitrogen dioxide (NO_2_) were both positively associated with purchasing patterns for coughs and colds, Hayfever and pain relief. Sulphur dioxide (SO_2_) was negatively associated with coughs and colds, and Hayfever. Age only appeared in the top five once and was negatively associated to sun preps. Across all the models 6 features rank in the top 10. 8 features rank in the top 10 for all models except sun preps, showing consistency important features across all product groups.

## Discussion

Loyalty card records from a national high street retailer have provided intriguing findings about self-medication patterns in England via novel application of machine learning. The large sample size of national level data on objective behaviours provides new context for customer behaviour of purchasing medicine, building on previous studies that have relied on small self-reported samples from specific regions that may be biased or less applicable to national-level decision making.

Our findings demonstrate that coughs and colds and pain relief medicines both have high proportions of purchasing, representing their common prevalence as minor ailments, with median proportions per LAD above 55%. Sun preps were the least common medication purchased, particularly for males. There are numerous potentially explanations for this. One explanation is that females are more likely to be responsible or informed about the adverse effects of the sun, and therefore engage in protective measures [36]. Such differences may account for skin cancer rates being higher in males. Targeting males through loyalty card records may offer one approach for tackling such patterns. That being said, sun prep purchasing patterns are far lower than self-reported estimates from other surveys [37], which may represent their bias or that individuals purchase sun preps from other locations as well. Another possible explanation is that Sun Preps are solely preparatory whereas the other medicines can serve as response to immediate discomfort. There may influence people purchasing for their household and despite physically purchasing a product they may not actually consume the product, particularly in the instance of families. Surprisingly, we detect little difference between genders for the other medicines which contrasts to the wider literature demonstrating females having higher likelihood of consuming non-prescribed medicines [21,38].

We detect considerable geographical inequalities in purchasing patterns for each of our medications. A North-South divide is highlighted, with the distribution of purchasing patterns following the known distribution of socio-economic measures and in particular poverty/deprivation [39]. This observation extends to southern population centres clearly highlighted having the higher proportions of purchasing, and in particular the suburban surrounds of London. Our data offers potential for geographic targeting of locations to increase self-medication behaviours. Sun preps once again differ in their distribution, with higher purchasing in urban and central regions of England. It is important to note that purchasing behaviours were lowest in coastal regions, which have been found to have higher UV radiation levels compared to inland locations [40]. These areas though are also characterised by older populations and given that purchasing behaviours for sun preps declined with age (Fig 4) this may also explain our findings. Given the difference in protective behaviours and risk of skin cancers, these represent important areas to focus targeting of interventions.

Socio-economic features were consistently shown to be associated with the purchasing of each medicine. IMD Score is consistently important in all models, exhibiting a negative association. For pain relief, aspiring and affluent OAC group has a positive spike between 0 and .1 with a slight positive correlation observed. Challenged Asian terraces OAC group are shown negatively correlated. The OAC pen portraits describes a group that exhibits high unemployment and overcrowding [41]. These findings follow previous research which has found positive associations between higher socioeconomic status and OTC usage [21,38]. These associations link to income and education levels associated with such occupations. Individuals with higher levels of income have greater disposable resources that can be invested in purchasing self-medications. Increased educational attainment may also represent greater cognitive resources and therefore greater awareness towards understanding how or the need to self-treat ailments [12]. The socio-economic findings, particularly IMD score, show a correlation between deprivation and decreased proportion of purchasing OTC products.

Age was identified as an important feature in the sun preps model. The partial dependency plot shows a negative association with age. Potential causes are that protection against the sun declines with age, with younger ages representing customers purchasing for dependent others i.e. mothers protecting their children against sunburn, or lower compliance with medicine guidelines as age increases [42,43]. Targeting older individuals who may be at risk of sunburn and skin cancer represents an important focus for policy makers.

Air quality was found to be an important contextual predictor of purchasing behaviours for all products other than sun preps (given there is little causal expectation of such a relationship for sun preps, this was expected). PM10 and NO2 are shown to be positively correlated with purchasing in the coughs and colds, Hayfever and pain relief models. This relates to rates being higher in urban areas resultant of transport [27,44–46]. PM10 exhibiting high feature importance as well as a positive correlation with increased levels aligns with research that exposure to traffic-related air pollution is associated with increased risk of Allergic Rhinitis (Hayfever) and reduced lung function (which may make individuals more susceptible to respiratory issues such as coughs and colds) [46]. SO2 distribution in the partial dependency plots is unconventional being negatively correlated to purchasing behaviours, then increasing and levelling off. SO2 is considered harmful at high concentrations, and such levels are often found in areas of intense industry which are typically not urban [47]. Similar to pollution, major conurbations (RUC) exhibits a positive association for cough and colds, possibly linked to the ailments typically being viral.

There are several limitations to our study. The data agreement signed by the high street retailer means that sample characteristics must remain anonymous. This constrains our ability to report on how representative the data are, a necessary component of any research. Despite the inclusion of 50 features, the study only utilises a select group of variables limiting the exploration to purely socio-economic and environmental characteristics. Data linkage could identify further knowledge, such as Hospital Admission data or even open prescription data, although information could only be linked at geographic scale as individuals are anonymised. In this study, we consider only whether someone has purchased a product within the 2-year period. Involving temporal aspects could aid further understanding. This approach could see further data from weather stations involved to see if there are seasonal effect apparent. The limitation of not knowing who the individuals are purchasing for–themselves or significant others–means that the results are purely based on purchasing and demand side factors. We are also unaware of actual usage of products. Our analyses are also cross-sectional and are limited in their ability to draw inferences about relationships to sociodemographic variables. There are also ecological fallacies and inferences about how they apply towards understanding individual-level relationships that should be avoided.

## Conclusion

This research utilises big data giving an understanding of large sample purchasing behaviour. The data contains close to 20% of the adult population in England, far larger than any previous self-medication study. The data driven approach using loyalty card data allows for actual purchasing behaviour captured within the data, allowing unprecedented context within data. This approach is a novel contribution to current self-care debate, hopefully allowing for further research expanding on the findings.

## Supporting information

S1 Table Variables included in machine learning and their source. (DOCX)
