# Supplementary material for: Using machine learning to investigate self-medication purchasing in England via high street retailer loyalty card data
Source: PLoS One. 2018 Nov 19;13(11):e0207523. doi: 10.1371/journal.pone.0207523 (PMC6242371; doi:10.1371/journal.pone.0207523)
Supplement: S1 Table — (DOCX) [file pone.0207523.s001.docx]

**S1 Table. Variables included in machine learning and their source**

| **Variable** | **Resource** |
| --- | --- |
| Age | High Street Retailer via CDRC (Median Age aggregated) |
| AHAH (components: Dentists; Emergency Departments; Fast Food; Gambling; GP Practices; Greenspace 900m; Leisure; NO2; Off Licences; Pharmacies; PM10; Pubs; SO2; Tobacconists) | CDRC (Access to Healthy Assets and Hazards) |
| IMD Score | Gov.uk (Index of Multiple Deprivation) |
| Rural-Urban Classification | Nomis (ONS Census Key Statistics) |
| Output Area Classification | ONS via CDRC (2011 Output Area Classification Geodata pack) |
